# Enhancing β-Galactosidase Performance for Galactooligosaccharides Preparation via Strategic Glucose Re-Tunneling

**DOI:** 10.3390/ijms252212316

**Published:** 2024-11-16

**Authors:** Jihua Zhao, Dandan Niu, Jiaqi Liu, Zhuolin Jin, Nokuthula Peace Mchunu, Suren Singh, Zhengxiang Wang

**Affiliations:** 1 Department of Biological Chemical Engineering, College of Chemical Engineering and Materials Science, Tianjin University of Science and Technology, Tianjin 300457, China; zhaojihua26@163.com (J.Z.); 22811991@mail.tust.edu.cn (J.L.); jzl1282803829@163.com (Z.J.); np.mchunu@nrf.ac.za (N.P.M.); 2 National Research Foundation, Pretoria 0001, South Africa; 3 School of Life Science, University of KwaZulu Natal, Durban 4000, South Africa; 4 Department of Biotechnology and Food Science, Faculty of Applied Sciences, Durban University of Technology, Durban 4001, South Africa; singhs@dut.ac.za; 5 Tianjin Key Laboratory of Industrial Microbiology, College of Biotechnology, Tianjin University of Science and Technology, Tianjin 300457, China

**Keywords:** β-galactosidase, re-tunneling glucose relocation, catalytic efficiency, galactooligosaccharides

## Abstract

This study focuses on the characterization and re-engineering of glucose transport in β-galactosidase (BglD) to enhance its catalytic efficiency. Computational prediction methods were employed to identify key residues constituting access tunnels for lactose and glucose, revealing distinct pockets for both substrates. In silico simulated saturation mutagenesis of residues T215 and T473 led to the identification of eight mutant variants exhibiting potential enhancements in glucose transport. Site-directed mutagenesis at T215 and T473 resulted in mutants with consistently enhanced specific activities, turnover rates, and catalytic efficiencies. These mutants also demonstrated improved galactooligosaccharide (GOS) synthesis, yielding an 8.1–10.6% enhancement over wild-type BglD yield. Structural analysis revealed that the mutants exhibited transformed configurations and localizations of glucose conduits, facilitating expedited glucose release. This study’s findings suggest that the re-engineered mutants offer promising avenues for enhancing BglD’s catalytic efficiency and glucose translocation, thereby improving GOS synthesis. By-product (glucose) re-tunneling is a viable approach for enzyme tunnel engineering and holds significant promise for the molecular evolution of enzymes.

## 1. Introduction

Enzymes, extremely important biocatalysts in biological systems, are integral to facilitating biochemical reactions both within living organisms and across various industrial sectors, including biomanufacturing, food production, textiles, pharmaceuticals, chemical processing, and environmental management [1,2,3,4]. These biocatalysts are produced by living systems, often genetically engineered, at scale, and with cost-effective operational expenditures [5].

Enzymes are recognized as natural catalysts that speed up the rate of chemical reactions by lowering their activation energy [6]. Achieving high catalytic efficiency under optimal conditions is a pivotal objective within the field of enzyme engineering [7]. Their catalysis rate is influenced not only by extrinsic factors such as pH, temperature, and substrate concentration but also by the inherent structural features of the enzymes themselves. Strategic alterations, either through site-directed or random mutagenesis of amino acid residues, can induce microstructural modifications that result in the artificial evolution of enzymes, enhancing their catalytic rates to meet industrial application demands [8].

β-Galactosidases, also known as lactases (EC3.2.1.23), were initially characterized by their ability to hydrolyze lactose into glucose and galactose [9,10]. Recent studies have revealed that, beyond lactose hydrolysis, β-galactosidases possess various other functions [11]. Notably, their capability to synthesize galactooligosaccharides (GOS), which serve as important prebiotics, has been discovered and has garnered industrial interest [12]. Currently, the most representative β-galactosidases suitable for GOS production are derived from *Aspergillus oryzae* [13] and *Bacillus circulans* [14]. The β-galactosidase from *A. oryzae* exhibits significant hydrolytic activity toward lactose. Therefore, specialized techniques and continuous in vitro evolution of the enzyme have been implemented for GOS production from lactose. An intelligent hydrophobic amino acid scanning strategy was used to reshape the active site of β-galactosidase to improve GOS synthesis efficiency and yield [15]. The β-galactosidase from *B. circulans* (BglD) is currently the most effective enzyme for GOS production due to its predominant galactosyl transfer activity and minimal hydrolytic activity, which prevents the formation of free galactose in the reaction mixture [12,16]. Given the low enzymatic activity of BglD produced by the *B*. *circulans* strain, enhancing its catalytic efficiency, in addition to achieving high-level expression, represents an effective strategy to improve its production efficiency and further benefit its application in GOS synthesis.

Apart from a few enzymes whose catalysis occurs on the outer surface of the enzyme, the active site of most enzyme molecules is located within a specific region inside the enzyme molecule, often in a pocket or cleft that provides a unique environment for the chemical reaction to occur [17]. The structural intricacies of enzymes, such as tunnel and channel formations, are recognized as pivotal elements that facilitate substrate ingress and product egress, thereby optimizing catalytic efficiency [18,19,20]. These structures not only serve as pathways to the active site but also minimize side reactions and potential inhibitory effects.

This study hypothesizes that the retention of glucose within the β-galactosidase’s (BglD) channel inhibits the release of the enzyme-product complex, thereby affecting the enzyme activity and further hindering the conversion of lactose to GOS. By altering the binding site of BglD to glucose, it is proposed that glucose release could be expedited, thus enhancing the catalytic rate. The investigation employs molecular docking technology, beginning with an analysis of substrate and product channels within the β-galactosidase enzyme, followed by simulated saturation mutagenesis, and molecular docking to identify optimal mutation sites and mutant candidates. Mutants were obtained by site-directed mutagenesis and heterologous expression and were comprehensively analyzed for specific enzyme activities, kinetic parameters, GOS conversion rates, and glucose channel characteristics. This research elucidates that facilitating side-product channel clearance can significantly boost the catalytic efficiency and specific activity of β-galactosidase and further increase the synthesis yield of GOS, offering a promising strategy for improving the catalytic performance of other enzymes as well.

## 2. Results

### 2.1. Characterization of Putative Glucose Pockets and Channels in β-Galactosidase BglD

**Figure 1:**
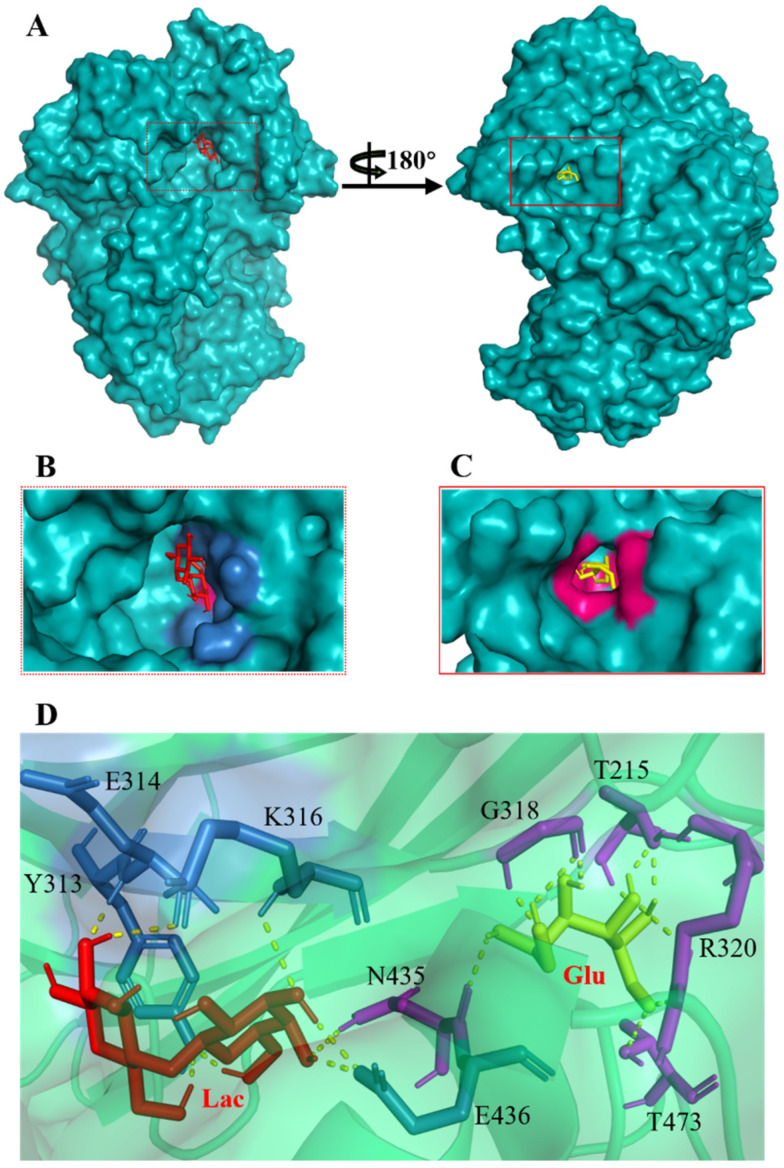
Detailed binding interactions of lactose and glucose with β-galactosidase BglD. (**A**) Comparative overview of BglD’s structure with bound lactose (left) and glucose (right). (**B**) Enlarged view of the lactose-binding site. The red stick was the lactose molecule and the blue part was the amino acid residues docked with lactose. (**C**) Enlarged view of the glucose-binding site. The yellow stick was the glucose molecule and the magenta part was the amino acid residues docked with glucose. (**D**) Close-up on the microenvironment facilitating the binding of lactose and glucose. The amino acid residues involved in binding lactose included Y313, E314, K316, N435, and E436, while glucose binding involved T215, G318, R320, N435, and T473. Notably, N435 was a shared residue in the binding sites of both sugars.

The investigation of access tunnels within β-galactosidase BglD is critical for understanding the molecular transport mechanisms of ligands from the enzyme’s active sites to the external environment. We therefore employed computational prediction methods to delineate the residues that constitute the access tunnels for lactose and glucose in BglD (Figure 1A). A distinct “pocket” on the surface of BglD that interacts with lactose was illustrated (Figure 1B), which exhibits structural resemblance to the “pocket” at the catalytic active center. These findings suggest that this “pocket” may be intricately linked to the enzyme’s catalytic center and potentially function as conduits for substrate ingress. Additionally, the glucose-binding site also features a “pocket” that is postulated to be instrumental in the facilitation of glucose translocation and subsequent release (Figure 1C). A closer look at the microenvironment that promotes lactose and glucose binding, with key lactose-binding residues as T313, E314, K316, N435, and E436 and the glucose-binding residues as T215, G318, R320, N435, and T473, with N435 being a shared residue, were finally identified in both binding scenarios (Figure 1D). The strategic positioning of residues T215 and T473 at the entrance of the glucose “pocket” underscores their importance in glucose channeling. It is hypothesized that mutations at these loci could modulate glucose binding affinity, potentially enhancing the rate of glucose transport within the enzyme’s channel and, by extension, its catalytic turnover. Therefore, T215 and T473 emerge as prime targets for enhancing β-galactosidase’s catalytic efficiency through site-directed mutagenesis, a hypothesis supported by our analytical data. Another residue, R320, was suggested to be located at the entrance based on structural analysis and predictions. However, subsequent experiments indicated that the enzyme no longer retained its original catalytic function following the mutation of residue R320. The causes and mechanisms underlying this phenomenon remain unclear. Future studies will aim to further explore the role and significance of R320.

### 2.2. In Silico Analysis of Potential Sites for Redirecting Glucose Transport

Subsequent to the tunnel characterization, residues T215 and T473 of BglD were selected for in silico simulated saturation mutagenesis to explore potential enhancements in glucose transport. These residues were computationally mutated, and the resulting variants were analyzed for their interaction with glucose. Selection criteria for promising mutants were stringent, focusing on variants that exhibited a reduced binding energy with glucose relative to BglD, presented alterations in glucose-binding residues, and maintained a spatial distinction from the lactose-binding site. Adhering to these criteria, eight mutant variants, T215C, T215H, T215N, T215Y, T473L, T473S, T473V, and T473Y, were identified and selected as satisfying all conditions (Table 1). The results of docking glucose show more differences; the binding energy of the BglD was −3.68 kcal·mol^−1^; the binding energy of the T215 mutants were −2.39 to −2.91 kcal·mol^−1^, while that of the T473 mutants were weaker (−1.86 to −2.33 kcal·mol^−1^) (Table 1). The glucose-binding site of the mutants were on the protein surface, except T215Y. The binding sites of T215C, T215H, T215N, and T473Y were near the lactose-binding pocket, while the other mutants were far away from the lactose-binding pocket. These variants represent viable candidates for experimental validation, offering potential avenues for enhancing the enzymatic processing of glucose.

### 2.3. Enhanced Specific Activity and Catalytic Efficiency of BglD via Re-Engineered Glucose Translocation

Site-directed mutagenesis was utilized to introduce targeted mutations at the T215 and T473 residues in BglD, as hypothesized in earlier sections of this study. The mutants thus produced were subjected to expression and subsequent isolation, achieving purification as confirmed by the single band on SDS-PAGE (Figure S1).

Specific activity is a measure of the catalytic efficiency of an enzyme relative to its protein content. When an enzyme mutant exhibits enhanced specific activity, it often suggests that the mutant enzyme is more efficient at catalyzing the reaction per unit of protein compared to the wild-type enzyme [21]. Analysis of the specific activities of a total of eight mutants demonstrated consistent enhancements (Table 2), as predicted by the molecular evolution methodology articulated within this research (Table S1). Notably, the T215 and T473 series mutants showed increases in specific activity of approximately 54–96% and 79–85% (Table 2), respectively.

Their catalytic kinetic characteristics were further analyzed, and the results are compiled in Table 3. Under optimal conditions, these mutants exhibited significant improvements in turnover rates (*k*_cat_) and catalytic efficiencies (*k*_cat_/*K*_m_), with certain variants such as T215Y, T473L, and T473V displaying decreased Michaelis constants (*K*_m_) indicative of higher affinity for their substrates. Compared to BglD, the catalytic efficiency of the mutants increased by 43% to 216% (Table 3), indicating an enhanced catalytic rate for synthesis of lactose to GOS. Meanwhile, the glucose inhibition constants (*K*_i_) increased by 13.4% to 62.5%, indicating that glucose has a reduced inhibition effect on that activity of the mutants. Based on these experimental results, it can be concluded that the rate at which the by-product glucose transitions from its bound state with the enzyme to its free (released) state is the principal limiting factor of this enzymatic reaction. Modifying the internal glucose-binding sites of the enzyme to enhance glucose release proved to be an effective strategy for improving its catalytic efficiency.

### 2.4. Enhanced Efficacy and Quality in GOS Synthesis via Re-Engineered Glucose Relocation Mutants

**Figure 2:**
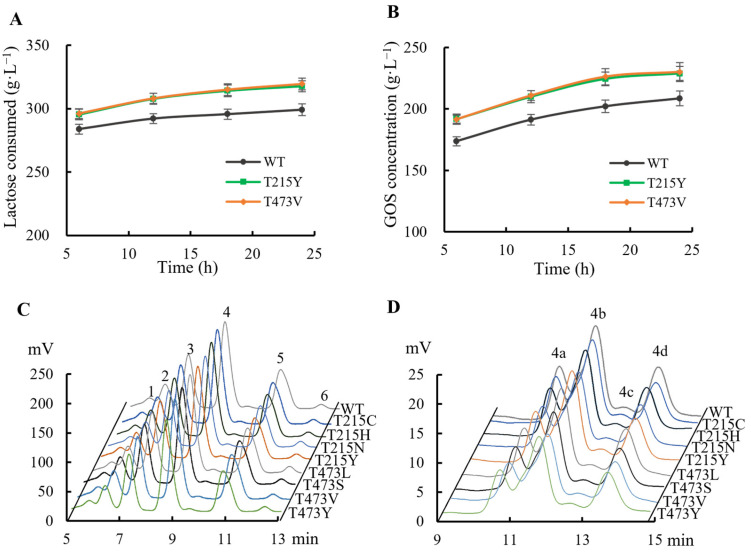
HPLC analysis of the variations in GOS synthesis mediated by BglD mutants. (**A**) GOS synthesis. (**B**) Lactose consumption. (**C**) Comparative chromatograms of the main components in the reaction mixture after 24 h incubation with different mutants. Peaks represented: 1, glucosylgalactotetraose or galactopentaose (DP5); 2, glucosylgalactotriose or galactotetraose (DP4); 3, glucosylgalactobiose or galactotriose (DP3); 4, disaccharide (DP2); 5, glucose; 6, galactose. (**D**) Detailed chromatogram of DP2 in 24 h–reaction mixture. Peaks 4a: galactobiose; 4b: lactose; 4c: allolactose (β(1→3)); 4d: allolactose (β(1→6)). The reactions were conducted using 2.4 U·mL^−1^ of the purified enzyme (BglD or its mutants) and 400 g·L^−1^ lactose in 100 mM sodium phosphate buffer (pH 6.0) at 50 °C and 120 rpm for 24 h. Samples were taken periodically during the incubation for HPLC analysis to monitor the changes in the mixture’s components.

Using the engineered mutants to mediate the synthesis of GOS, HPLC sugar profile analysis was conducted on the characteristics of its product composition and its time-course changes. The results are summarized in Figure 2 and Table 4. The mutants showed increased rates of GOS production and lactose consumption (Figure 2A,B and Table 4). Additionally, comparative HPLC profiles of the enzymatic mutants closely resembled those of BglD, as indicated in Figure 2C,D, being quantifiable and comparable across all variants (Table 4). These findings suggest that their transglycosylation function is slightly but not significantly increased in comparison to that of wild-type BglD, preserving the essential transglycosylation capability for GOS production [9,22]. The engineered enzymes facilitated an increase in the abundance of GOS components glucosylgalactobiose or galactotriose (DP3), glucosylgalactotriose or galactotetraose (DP4), and glucosylgalactotetraose or galactopentaose (DP5) (Figure 2C), achieving a total GOS yield of 56.3–57.6% (g/g total lactose) (Table 4), marking an 8.1–10.6% enhancement over BglD yield (Table 4).

Furthermore, this study observed a significant reduction in residual lactose in the reaction mixtures of the mutants (Figure 2A,B and Table 4), indicating an improved resistance to glucose inhibition and more efficient lactose utilization. All the mutants demonstrated higher lactose consumption rates than BglD while maintaining the original high ratio of transglycosylation/hydrolysis, thereby promoting GOS synthesis and leading to an enhanced lactose-to-GOS conversion yield of 2.4–3.3%. Additionally, the rate of GOS production and lactose consumption in the mutants were enhanced to 23.5–24.0 mg·g^−1^·h^−1^ and 32.8–33.3 mg·g^−1^·h^−1^, respectively, which increased by 8.3–10.6% and 5.1–6.7% compared with BglD (Table 4), supporting the augmented catalytic efficiency of the mutant enzymes.

### 2.5. Elucidation of Enhanced Catalytic Activity Through Structural Analysis

**Figure 3:**
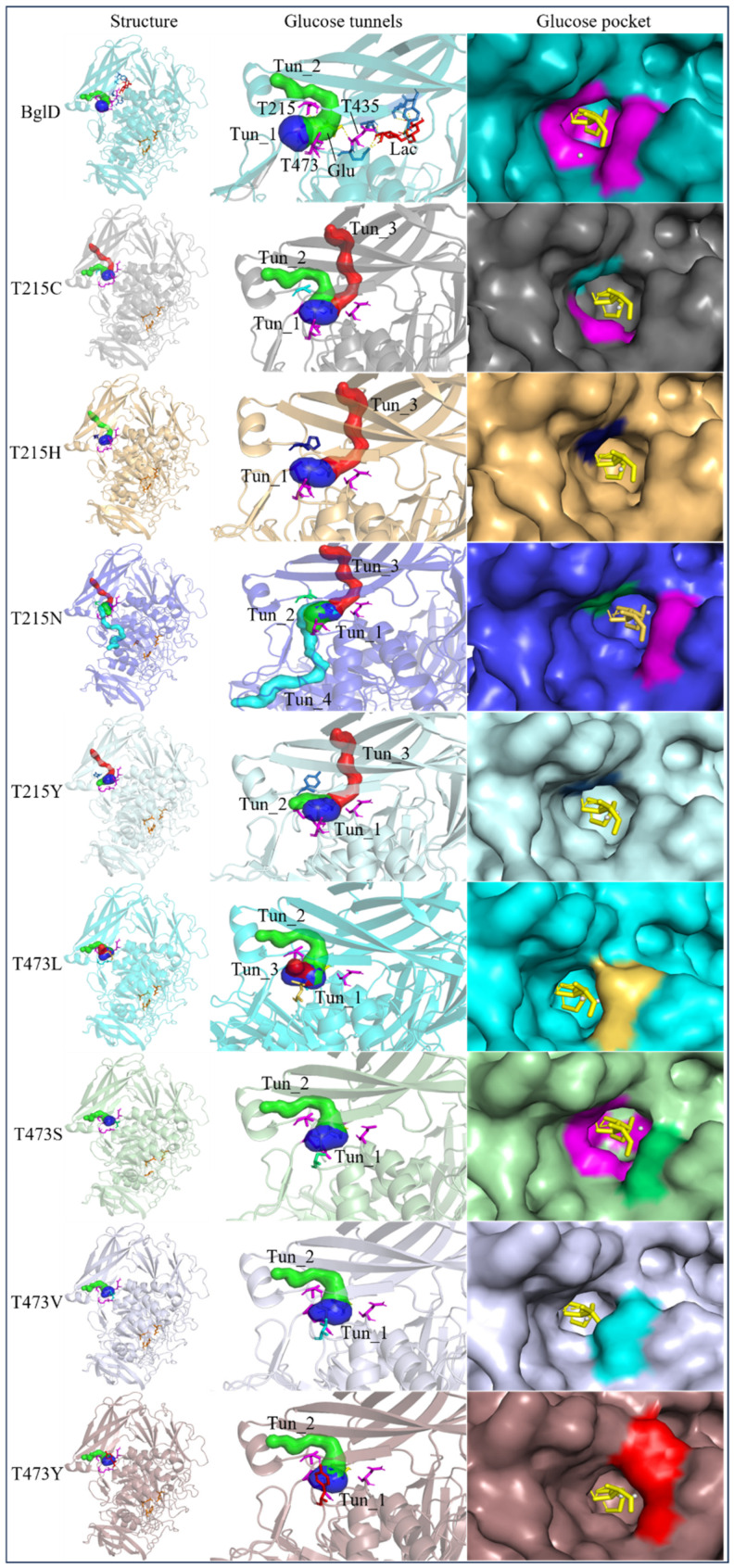
Structural changes following relocation of by-product glucose in BglD molecule. Left: Overview of the enzyme’s structure highlighting the location of the glucose tunnel. Middle: Detailed view of the glucose tunnel showcasing its alterations; Tunnel 1 (tun_1) is depicted in blue, Tunnel 2 (tun_2) in green, Tunnel 3 (tun_3) in red, and Tunnel 4 (tun_4) in cyan. Right: The glucose-binding pocket.

In pursuit of understanding the molecular underpinnings responsible for the amplified catalytic activity and efficiency, we examined the three-dimensional configurations of the T215 and T473 mutants utilizing a BglD crystal structure as a reference template and delineated the glucose release pathways with the aid of CAVER Web v1.2. Within the BglD structure, we identified two tunnel-like features, designated as tun_1 and tun_2, which exhibited substantial congruence (Figure 3). Subsequent molecular docking analyses revealed that the amino acid residue T435 served as a pivotal junction for the binding of glucose and lactose molecules, with the glucose moiety partially penetrating tun_2 (Figure 3). It was postulated that the glucose generated via the catalytic process traversed through residue T435, culminating in its egress through tunnels tun_1 and tun_2. Notably, alterations in the mutants manifested as transformed configurations and localizations of the glucose conduits in comparison to the BglD counterpart. Specifically, the entrance of tun_1 in the mutants penetrated deeper into the enzyme’s structure, facilitating the insertion of glucose into this channel (Figure 3). This structural modification likely contributes to the expedited release of glucose in the mutant enzymes [18,23,24].

Further comparative analysis of the tunnel structures between BglD and the mutants revealed an expansion in the bottleneck diameter of glucose tun_1 in the mutants, ranging from 2.1 to 2.3 Å, in contrast to the BglD’s 2.0 Å, with the exception of T473V (Table S1). Concurrently, the glucose tunnel’s “binding pocket” was observed to be more capacious (Figure 3), a structural enhancement typically associated with improved product (glucose) release efficiency [20,25]. Augmented efficiency in product discharge is often indicative of heightened enzymatic activity and catalytic proficiency. By broadening the channel, Kong et al. [26] successfully managed to decrease the resistance encountered during product release, thereby increasing the overall enzymatic activity. This study’s findings are in alignment with these observations, suggesting that the structural modifications in the mutants facilitate a more efficient catalytic cycle by reducing the impediments to product release.

Molecular docking results showed that the binding energy is indicative of the receptor–ligand interaction strength, and the stability of the resultant complex was found to be inversely proportional to the specific activities of the mutants, with T473 variants demonstrating weaker binding energies to glucose and consequently higher specific activities. The weaker binding energy indicated that the ligand glucose requires less energy to leave the protein, another indication that changes in the glucose tunnel made glucose release more efficient.

## 3. Discussion

In the present study, we have methodically developed a set of β-galactosidase mutants to advance the biosynthesis of GOS (Table 4 and Figure 2). This development is underscored by a pronounced increase in both the specific activity and the catalytic efficiency of the enzyme, with these improvements largely ascribed to strategic molecular interventions. These achievements were carefully designed to re-engineer the glucose pocket of BglD (Figure 3), consequently expediting the extrication of glucose as a by-product. The meticulously executed molecular alterations have been shown to significantly bolster the enzyme’s substrate affinity and reduce by-product inhibition, as evidenced by a reduced Michaelis constant (*K*_m_) and increased glucose inhibition constant (*K*_i_), and to amplify substrate turnover rates (Table 2 and Table 3). Altogether, these refinements have resulted in an enzymatic process that is markedly more efficient and precise [27].

Product inhibition is a well-documented phenomenon in enzyme catalysis, where the accumulation of reaction products can interfere with the enzyme’s activity, either by directly affecting the accessibility of the active site or by altering the enzyme’s structural stability [28]. In β-galactosidase-catalyzed reactions, glucose, a side-product, can lead to non-competitive inhibition. This form of inhibition does not involve direct competition at the active site; instead, glucose interferes with the release of the enzyme-product complex, which can hinder the conversion of lactose into GOS (Figure 3). The development of β-galactosidase mutants that can effectively circumvent such inhibitory effects, by displacing glucose from the vicinity of the glucose tunnel and the active site, represents a significant advancement in improving the enzyme’s specific activity and the overall kinetics of the reaction [29,30].

The substantial increase in catalytic efficiency observed in BglD mutants can be directly linked to kinetic enhancements resulting from a more accessible active site, discernible enlargement of glucose release channels, and expansion of the binding pocket dimensions (Table 3, Figure 3). Such structural refinements are posited to precipitate a decrease in the residence time of glucose within the enzyme’s confines, thereby expediting the catalytic cycle and culminating in an elevated turnover rate (Table 3, Figure 3). By strategically directing glucose away from the active site, the enzyme is better positioned to interact with incoming lactose molecules, enhancing the catalytic lactose process, which in turn promotes an increased production of GOS (Table 4, Figure 2). This kinetic improvement is in harmony with the principles of the push–pull hypothesis in enzyme kinetics. This hypothesis posits that the rapid removal of a product (push) from the reaction milieu can significantly elevate the reaction rate by inducing a shift in the equilibrium dynamics, favoring more pronounced product formation (pull) [31]. This concept aligns with the notion that push–pull mechanisms, absent of feedback, can maximize information transfer and align output with variable input in signaling systems, as demonstrated in other biochemical contexts [32].

This study underscored the boundless potential inherent in the rational design of enzymes as a means to refine and augment biocatalysts for industrial application. By exploring into and subsequently manipulating the structural determinants that are pivotal to enzyme functionality, the engineering of bespoke biocatalysts that boast unparalleled efficiency and productivity becomes a tangible reality [33]. Such advancements are of critical importance within the ambit of the competitive industrial biotechnology sector [34].

## 4. Materials and Methods

### 4.1. Strains, Plasmids, and Primers

The plasmid pNB-BglD, which harbors the β-galactosidase gene (the gene with GenBank accession number MN615268 was used as a reference) from *B. circulans* strain B2301 as detailed in prior studies [10,35], served as the DNA template for the manipulative procedures. The expression vector pHY-WZX [36] was employed in *Bacillus genus* host strains to facilitate the secretion and expression of β-galactosidase and its variants. *Escherichia coli* JM109 functioned as the cloning host, while *Bacillus licheniformis* CBBD302 [37] was utilized for protein expression. Cultivation of the strains was conducted at 37 °C and 220 rpm for 12–16 h in Luria–Bertani (LB) broth or LB plates at 37 °C. The media were supplemented with 100 µg·mL^−1^ ampicillin or 20 µg·L^−1^ kanamycin when required. Oligonucleotide sequences designed for site-directed mutagenesis are enumerated in Table 5.

### 4.2. Prediction and Structural Analysis of β-Galactosidase Mutation

Homology modeling of the three-dimensional structure for both the BglD and the mutants was performed using the SWISS-MODEL online service [38,39] (SWISS-MODEL Interactive Workspace (expasy.org)), utilizing the crystal structure of *B. circulans* ATCC 31382 (BgaD) (PDB: 4ypj.1) [40] as a reference template (93.6% sequence identity). The docking procedures were carried out using the AutoDock 4.2 software package [41], and the resulting docking data were visualized and reviewed using the PyMOL Molecular Graphics System [42].

For the molecular docking, glucose was employed as a ligand with β-galactosidase via the AutoDock software. The enzyme–glucose complex exhibiting the highest binding energy was chosen for in-depth analysis. Concurrently, molecular docking of β-galactosidase with lactose informed the selection of amino acid residue sites for enzyme–glucose binding. These amino acid residue sites were computationally saturated mutated to generate the proposed mutants, followed by molecular docking with glucose. The docking results were then scrutinized to identify prospective mutants with significant research potential.

To analyze the glucose access tunnels, the calculations were performed using the wild-type BglD crystal structure (PDB ID: 4YPJ.1) employing the CAVER Web v1.2 tool [43], available at https://loschmidt.chemi.muni.cz/caverweb/ (accessed on 10 January 2023). The starting point for the CAVER analysis was the position of glucose from the molecular docking results. The parameters for the CAVER calculations were set as follows: a minimum probe radius of 0.9 Å to define the narrowest passageway accessible to the glucose molecule, a shell depth of 4 Å to specify the extent of the tunnel’s surrounding area, a shell radius of 3 Å to delineate the width of the tunnel’s immediate environment, a clustering threshold of 3.5 Å to group similar tunnel trajectories, a maximum distance of 3 Å to limit the length of the tunnel segments considered in the analysis, and a desired radius of 5 Å to define the preferred width of the tunnel for glucose passage.

### 4.3. Site-Directed Mutagenesis of BglD

The site-directed mutagenesis of the β-galactosidase BglD gene was performed using a primer-mediated overlapping PCR technique as described previously [44]. The gene encoding β-galactosidase was amplified using pNB-BglD [35] as a template. The mutated genes encoding β-galactosidase were cloned into pHY-WZX [36] and introduced into *E. coli* JM109 by heat shock transformation [45], followed by subsequent transformation into *B. licheniformis* CBBD302 as previously described [37] for the preparation of β-galactosidase mutants. Verification of the mutation sites was accomplished through Sanger sequencing [46].

### 4.4. Expression and Purification of Mutants

The *B. licheniformis* CBBD302 transformants were cultivated at 37 °C with agitation at 200 rpm for 12–16 h in 50 mL of LB liquid medium. Subsequently, these cultures were inoculated into fresh 50 mL LB liquid medium at a 10% inoculum volume in a 250 mL Erlenmeyer flask and further incubated at 37 °C and 220 rpm for 72 h [36]. The supernatants were collected by centrifugation at 8000 rpm and 4 °C for 10 min. When necessary, the enzyme protein was isolated by precipitation using a saturated solution of ammonium sulfate at 50–60% saturation. The enzyme mutants underwent purification using an ÄKTA pure chromatography system (Cytiva, Uppsala, Sweden) equipped with a Superdex™ 200 Increase 10/300 GL column. The elution was performed with a 50 mM phosphate buffer at pH 7.2, maintaining a flow rate of 0.75 mL·min^−1^. The presence of β-galactosidase activity in the eluates was monitored, and fractions exhibiting activity were collected. The molecular weight and purity of the enzymes were assessed by SDS-PAGE, utilizing a 10% (*w*/*v*) resolving gel and a 5% (*w*/*v*) stacking gel [47], with reference to a protein molecular weight standard (#26,610, Thermo Fisher Scientific, Beijing, China). Protein concentrations were quantified using the Bradford assay [48], with bovine serum albumin fraction V (Roche Diagnostics GmbH, Mannheim, Germany) as the standard.

### 4.5. Enzyme Activity Assay and Kinetic Parameters

The activity of the purified β-galactosidase mutants was determined using lactose (Sigma-Aldrich, Shanghai, China) as substrate. The reactions were conducted at 40 °C in a 100 mM phosphate buffer (pH 6.0) containing 30 mM lactose and 0.5 μmol·L^−1^ of the purified enzyme. The reactions were halted after 20 min by boiling the mixture for 10 min, and the amount of glucose released was determined using a glucose oxidase biosensor (Biology Institute of Shandong Academy of Sciences, Jinan, China) [16]. One unit of enzyme activity is defined as the quantity of enzyme releasing one μmol of glucose per minute under the specified conditions. The ratio of transglycosylation (T) to hydrolysis (H) [T/(T + H)] (%) was calculated as the percentage of GOS content (g·L^−1^) relative to the total of GOS and galactose (g·L^−1^) in the reaction mixture [49].

The kinetic parameters, *K*_m_ and *k*_cat_, for the mutant enzymes were calculated using lactose concentrations ranging from 10 to 500 mM based on the previously described assay. Enzyme activity was measured by adding glucose (50 to 500 mM) to the reaction system, and the inhibition constant (*K*_i_) was calculated according to the *K*_m_,_app_ method [50]. The *k*_cat_ value was derived by dividing the *V*_max_ by the enzyme concentration. *V*_max_ and *K*_m_ were determined by fitting the initial rate data to the Michaelis–Menten equation using nonlinear regression. All experiments to ascertain enzymatic characteristics were performed in triplicate, with standard deviations represented as error bars.

### 4.6. Production of GOS from Lactose

The synthesis of GOS from lactose was performed in a 25 mL reaction vessel containing 400 g·L^−1^ lactose in 100 mM phosphate buffer (pH 6.0) and 2.4 U·mL^−1^ of the purified enzymes (BglD or its mutants). The reactions were carried out at 50 °C with stirring at 120 rpm for 24 h. Reactions were terminated by boiling the mixtures for 10 min. The quantification of GOS and other sugars (lactose, glucose, and galactose) was conducted using a HPLC system (Agilent 1260, Santa Clara, CA, USA) equipped with a refractive index detector (Shodex RI-201H, Shoko Science Co., Ltd., Yokohama, Japan) and dual columns [Sugar-Pak™ I column (Waters, Shanghai, China, 6.5 mm × 300 mm) and YMC-Pack NH2 (250 mm × 4.6 mm)]. The separation of sugars by degree of polymerization (DP) was achieved on the Sugar-Pak™ I column with Milli-Q water as the mobile phase at a flow rate of 0.5 mL·min^−1^ at 80 °C. Disaccharides such as galactobiose, allolactose, and lactose, which have identical retention times in this system, were further distinguished using the YMC-Pack NH2 column with 70% (*v*/*v*) acetonitrile as the mobile phase at an elution rate of 1 mL·min^−1^ at 35 °C. Calibration curves for lactose, glucose, and galactose, established through linear regression, were employed for quantification.

## 5. Conclusions

In the present investigation, we have systematically demonstrated that the accessibility of the glucose translocation pathway can be significantly improved by mitigating the binding affinity of glucose to the enzyme. This was achieved by strategically engineering variants of *B. circulans* β-galactosidase through an integrated approach encompassing homology modeling, molecular docking, and rigorous analysis of candidate mutation sites, followed by the judicious selection of promising mutants and the application of site-directed mutagenesis. Remarkably, these rationally designed mutants exhibited enhanced ability to reduce the residual lactose concentration in the reaction mixture, thereby facilitating the efficient catalytic production of GOS from lactose. The mutation strategy was effective in increasing the overall catalytic turnover of any reaction, both hydrolysis and transglycosylation. The findings from this study not only underscore the efficacy of the employed engineering strategy but also have profound implications for the advancement of enzyme channel engineering. Furthermore, the insights gleaned from this research contribute to the broader understanding of molecular evolution processes in enzymes with similar functionalities.

## Figures and Tables

**Table 1:** Analysis of structural characteristics of proposed mutants docked to glucose.

Enzyme	Binding Energy ^a^ (kcal·mol^−1^)	Binding Site ^b^	Distinction from Lactose-Binding Site ^c^
BglD	−3.68	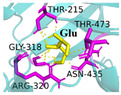	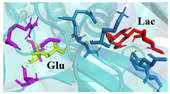
T215C	−2.62	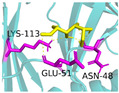	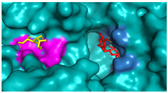
T215H	−2.39	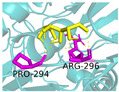	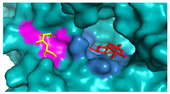
T215N	−2.42	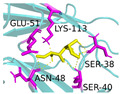	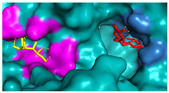
T473Y	−2.33	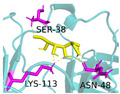	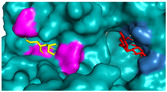
T215Y	−2.91	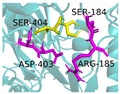	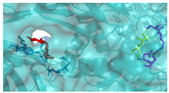
T473L	−2.26	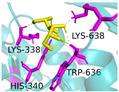	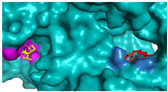
T473S	−1.86	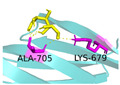	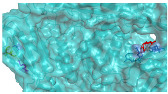
T473V	−1.95	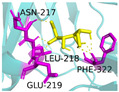	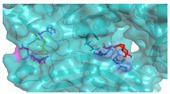

^a^ Binding energy of the selected mutant for docking with glucose molecules. ^b^ The binding site of the mutant to glucose. ^c^ Spatial distance and location between the glucose-binding site and the lactose-binding site. The red stick was the lactose molecule and the blue part was the amino acid residues docked with lactose. The yellow stick was the glucose molecule and the magenta part was the amino acid residues docked with glucose.

**Table 2:** Specific activity of BglD and mutants ^a^.

Enzyme	Specific Activity (U·mg^−1^)	Folds of Specific Activities Comparison to BglD
BglD	109.6 ± 2.6	1.00
T215C	169.2 ± 5.1	1.54
T215H	214.8 ± 5.8	1.96
T215N	145.2 ± 3.6	1.33
T215Y	162.3 ± 4.2	1.48
T473L	195.8 ± 5.1	1.79
T473S	200.7 ± 5.8	1.83
T473V	187.5 ± 5.2	1.71
T473Y	203.2 ± 4.3	1.85

^a^ Specific activity was determined at 40 °C in a 100 mM phosphate buffer (pH 6.0) containing 30 mM lactose.

**Table 3:** Kinetic parameters (mean ± standard deviation of triplicate experiments) of BglD and mutants ^a^.

Enzyme	*K*_m_ (mM)	*k*_cat_ (s^−1^)	*k*_cat_/*K*_m_ (s^−1^·mM^−1^)	*K*_i_ (mM)
BglD	118.1 ± 6.3	23.9 ± 1.3	0.20	84.8 ± 5.4
T215C	111.3 ± 5.0	55.5 ± 2.8	0.50	126.5 ± 6.8
T215H	114.5 ± 5.8	34.9 ± 1.9	0.30	137.8 ± 7.1
T215N	107.9 ± 4.0	38.0 ± 2.4	0.35	102.4 ± 5.2
T215Y	89.4 ± 6.4	50.9 ± 2.5	0.57	96.2 ± 4.6
T473L	52.7 ± 3.6	33.7 ± 2.1	0.64	112.5 ± 5.2
T473S	110.6 ± 6.5	50.0 ± 2.4	0.45	133.6 ± 7.0
T473V	84.6 ± 4.9	45.5 ± 1.4	0.54	131.6 ± 6.8
T473Y	114.2 ± 4.9	33.1 ± 1.1	0.29	117.4 ± 4.9

^a^ Kinetic parameters (*K*_m_ and *k*_cat_) were determined with 10 different lactose concentrations ranging from 10 to 500 mM at 40 °C and pH 6.0.

**Table 4:** Contents of each sugar component in the mutant-mediated synthesis of GOS ^a^.

Enzyme	Sugar Profile and Content in the Final Reaction Mixture ^b^ (g·L^−1^)			Lactose Consumed ^c^ (g·L^−1^)	GOS Yield (%) ^d^	Lactose-to-GOS Yield (%) ^e^	GOS Productivity ^g^ (mg·g^−1^·h^−1^)	Lactose Consumption Rate ^h^ (mg·g^−1^·h^−1^)	T/(T + H) (%) ^f^
	Lactose	Glucose	Galactose						
BglD	100.8 ± 2.8	82.6 ± 1.6	8.0 ± 0.3	299.2 ± 7.8	52.1 ± 1.0	69.7 ± 2.1	21.7 ± 1.3	31.2 ± 1.1	96.3 ± 0.2
T215C	83.9 ± 1.7	81.0 ± 1.5	7.8 ± 0.2	316.1 ± 8.0	56.8 ± 1.3	71.9 ± 2.6	23.7 ± 1.4	32.9 ± 1.3	96.7 ± 0.1
T215N	85.6 ± 1.8	80.6 ± 1.7	8.4 ± 0.3	314.4 ± 8.2	56.4 ± 1.7	71.7 ± 2.5	23.5 ± 0.9	32.8 ± 0.9	96.4 ± 0.1
T215H	82.3 ± 2.0	81.6 ± 1.8	9.3 ± 0.3	317.7 ± 8.5	56.7 ± 2.0	71.4 ± 2.0	23.6 ± 1.0	33.1 ± 1.0	96.1 ± 0.1
T215Y	82.2 ± 1.9	80.8 ± 1.7	8.4 ± 0.2	317.8 ± 7.5	57.2 ± 2.3	72.0 ± 1.9	23.8 ± 0.8	33.1 ± 1.3	96.5 ± 0.2
T473L	81.6 ± 2.3	81.7 ± 1.9	9.5 ± 0.3	318.4 ± 7.1	56.8 ± 1.8	71.4 ± 3.2	23.7 ± 1.1	33.2 ± 1.8	96.0 ± 0.1
T473S	81.1 ± 2.5	81.2 ± 1.8	8.8 ± 0.2	318.9 ± 5.9	57.2 ± 2.1	71.8 ± 2.1	23.8 ± 1.4	33.2 ± 1.1	96.3 ± 0.2
T473V	80.4 ± 2.5	81.3 ± 1.7	8.1 ± 0.1	319.6 ± 6.9	57.6 ± 2.5	72.0 ± 2.5	24.0 ± 1.2	33.3 ± 1.5	96.6 ± 0.2
T473Y	85.5 ± 1.8	80.8 ± 1.8	8.4 ± 0.1	314.5 ± 8.1	56.3 ± 1.9	71.6 ± 2.4	23.5 ± 1.4	32.8 ± 1.4	96.4 ± 0.1

^a^ GOS was produced using 2.4 U·mL^−1^ purified enzymes (BglD or its mutants) with 400 g·L^−1^ lactose [in 100 mM sodium phosphate buffer (pH 6.0)] incubated at 50 °C and stirred at 120 rpm for 24 h. ^b^ The concentrations of lactose, glucose, and galactose are calculated by peak intensities in the HPLC profiles. ^c^ Lactose consumed = initial lactose (g·L^−1^) − [remaining lactose (g·L^−1^) after 24 h]. ^d^ GOS yields are calculated as grams of GOS produced from 100 g of initial lactose. Calibration curves for lactose, galactose, and glucose ranging from 0.1 to 5 g·L^−1^ were used for quantification. ^e^ Lactose-to-GOS yield coefficients are calculated as grams of GOS produced from 100 g of consumed lactose. ^f^ The ratio of transglycosylation (T)/hydrolysis (H) [T/(T + H)] = the GOS concentration (g·L^−1^)/the concentration of GOS and galactose (g·L^−1^) × 100%. ^g^ GOS productivity is calculated as milligrams of GOS produced per hour from initial lactose per gram. ^h^ Lactose consumption rate is calculated as milligrams of lactose consumed per hour from initial lactose per gram.

**Table 5:** Oligonucleotides used in this study.

Primer	Nucleotide Sequence (5′ → 3′) *
BglD-F	GCTGGATCCAGCAAGACTACCTCCGCTGCTG
BglD-R	AGGAGTGACGGTGAAAACAGAAG
T215C-F	TTTCGTTACCTGCCCAAACTTGG
T215C-R	CCAAGTTTGGGCAGGTAACGAAA
T215H-F	TTTCGTTACCCATCCAAACTTGG
T215H-R	CCAAGTTTGGATGGGTAACGAAA
T215N-F	TTTCGTTACCAACCCAAACTTGG
T215N-R	CCAAGTTTGGGTTGGTAACGAAA
T215Y-F	TTTCGTTACCTATCCAAACTTGG
T215Y-R	CCAAGTTTGGATAGGTAACGAAA
T473L-F	GATTGACACCCTGAGACCTACCA
T473L-R	TGGTAGGTCTCAGGGTGTCAATC
T473S-F	GATTGACACCAGCAGACCTACCA
T473S-R	TGGTAGGTCTGCTGGTGTCAATC
T473V-F	GATTGACACCGTCAGACCTACCA
T473V-R	TGGTAGGTCTGACGGTGTCAATC
T473Y-F	GATTGACACCTATAGACCTACCA
T473Y-R	TGGTAGGTCTATAGGTGTCAATC

* Underlined nucleotide sequence encoded a mutated amino acid.

## Data Availability

Data are contained within the article and Supplementary Materials.

## Supplementary Materials

The following supporting information can be downloaded at: https://www.mdpi.com/article/10.3390/ijms252212316/s1.
