# Supplementary material for: Enhancing β-Galactosidase Performance for Galactooligosaccharides Preparation via Strategic Glucose Re-Tunneling
Source: Int J Mol Sci. 2024 Nov 16;25(22):12316. doi: 10.3390/ijms252212316 (PMC11594752; doi:10.3390/ijms252212316)
Supplement: Supplementary file 1 [file ijms-25-12316-s001.zip › ijms-3250811-supplementary.pdf]

Supplementary Materials:

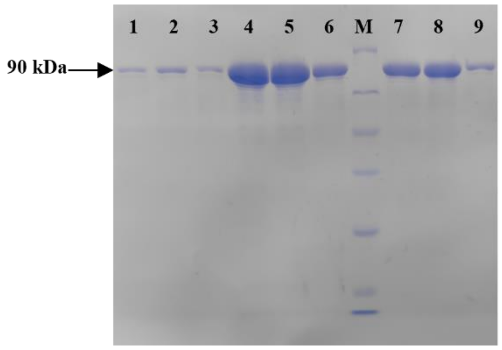

Figure S1. SDS-PAGE profile of purified enzymes. Lane 1: BglD; Lanes 2-9: Mutants T215C, T215H, T215N, T215Y, T473L, T473S, T473V, and T473Y, respectively; M: Protein molecular weight standard (116, 66.2, 45, 35, 25, 18.4, 14.4 kDa)

Table S1. Substrate access tunnel parameters calculated by CAVER Web.

Enzyme	Bottleneck radius(Å)[a]					Length(Å)[b]					Throughput[c]					Curvature[d]				
	Tun_1	Tun_2	Tun_3	Tun_4	Sum	Tun_1	Tun_2	Tun_3	Tun_4	Sum	Tun_1	Tun_2	Tun_3	Tun_4	Sum	Tun_1	Tun_2	Tun_3	Tun_4	Sum
BglD	2.0	0.9			2.9	1.9	28.0			29.9	0.92	0.27			1.19	1.0	2.4			3.4
T215C	2.3	1.0	0.9		3.3+0.9	4.4	22.4	25.1		26.8+25.1	0.89	0.25	0.22		1.14+0.22	1.1	1.7	1.5		2.8+1.5
T215H	2.3	0.9			3.2	4.9	24.8			29.7	0.89	0.24			1.13	1.2	1.5			2.7
T215N	2.3	1.3	0.9	0.9	3.6+0.9+0.9	3.9	8.2	24.3	52.3	12.1+24.3+52.3	0.88	0.72	0.25	0.06	1.6+0.25+0.06	1.1	1.5	1.5	1.8	2.6+1.5+1.8
T215Y	2.3	1.0	0.9		3.3+0.9	4.4	8.3	25.5		12.7+25.5	0.93	0.61	0.22		1.54+0.22	1.1	1.3	1.6		2.4+1.6
T473L	2.2	1.3	0.9		3.3+0.9	3.9	8.2	22.3		12.1+22.3	0.90	0.75	0.29		1.65+0.29	1.1	1.3	1.8		2.4+1.8
T473S	2.2	0.9			3.1	4.7	20.8			25.5	0.89	0.29			1.18	1.2	1.6			2.8
T473V	1.7	1.0			2.7	5.2	20.8			26.0	0.85	0.29			1.14	1.3	1.6			2.9
T473Y	2.1	0.9			3.0	2.4	22.4			24.8	0.93	0.29			1.22	1.1	1.8			2.9

[a] The bottleneck radius means that the narrowest part of a given access tunnel.
[b] The length of a given access tunnel.
[c] Throughout reflects the probability that the pathway is used as a route for transport of the substances.
[d] The curvature of a given access tunnel which is calculated as length/distance, where length is the length of the tunnel and distance is the shortest possible distance between the calculation starting point and the tunnel ending point.
